# Pharmacological screening of *Monotheca buxifolia* (Falc.) A. DC. for antinociceptive, anti-inflammatory and antipyretic activities

**DOI:** 10.1186/s12906-016-1257-z

**Published:** 2016-08-05

**Authors:** Irfan Ullah, Jamshaid Ali Khan, Muhammad Shahid, Ajmal Khan, Achyut Adhikari, Peer Abdul Hannan, Ibrahim Javed, Faisal Shakeel, Umar Farooq

**Affiliations:** 1 Department of Pharmacy, University of Peshawar, Peshawar, 25120 Pakistan; 2 Department of Pharmacy, Abasyn University, Peshawar, Pakistan; 3 Department of Chemistry, COMSATS Institute of Information Technology, Abbottabad, Pakistan; 4 HEJ Research Institute of Chemistry, International Centre for Chemical and Biological Sciences, University of Karachi, Karachi, Pakistan; 5 Department of Chemistry, SBA School of Science and Engineering, Lahore University of Management Sciences, Lahore, Pakistan

**Keywords:** *Sapotaceae*, Oleanolic acid, Isoquercetin, Pain, Inflammation, Pyrexia

## Abstract

**Background:**

Medicinal plants have proven their importance as a valuable source of molecules with therapeutic potential. *Monotheca buxifolia* (Falc.) A. DC. (family: *Sapotaceae*) is traditionally used as a hematinic, laxative, digestive, anthelmintic, antipyretic, and in the treatment of gastro-urinary disorders. To provide scientific evidence for its folkloric use, the present study investigated *Monotheca buxifolia* fruit hydro-ethanolic extract (MBHE) for its prospective antinociceptive, anti-inflammatory and antipyretic activities. MBHE was eluted through column chromatography to isolate the bioactive secondary metabolites which may probably involve in its beneficial properties.

**Methods:**

The phytochemical constituents in MBHE was elucidated using UV, IR, ^1^H-NMR, ^13^C NMR, 2D-NMR spectra in combination with EIMS and FAB-MS spectrometric techniques and comparison with literature data of related compounds. The antinociceptive activity of MBHE was evaluated in the acetic acid induced abdominal constriction assay; the anti-inflammatory potential was assessed in the carrageenan induced paw edema, while the antipyretic effect was tested against brewer’s yeast induced pyrexia in BALB/c mice at doses of 50, 100 and 150 mg/kg.

**Results:**

Elution of MBHE along with various characterization techniques led to the isolation of oleanolic acid and isoquercetin. Significant attenuation of chemical induced nociception was observed with MBHE at tested doses of 50 mg/kg (*P* < 0.01, 68.87 %), 100 mg/kg (*P* < 0.01, 68.87 %) and 150 mg/kg (*P* < 0.001, 83.02 %). During a duration of 1–5 h in the carrageenan induced paw edema assay, significant ameliorative effect (*P* < 0.01, *P* < 0.001) was demonstrated by MBHE at 50 mg/kg (22.94–20 %), 100 mg/kg (33.23–21.13 %) and 150 mg/kg (38.23–25 %). MBHE also significantly alleviated the brewer’s yeast induced pyrexic response when tested at doses of 50 mg/kg (*P* < 0.05 in 2nd h), 100 mg/kg (*P* < 0.05, *P* < 0.01 and *P* < 0.001 in 1–5 h) and 150 mg/kg (*P* < 0.01 and *P* < 0.001 in 1–5 h).

**Conclusion:**

These findings suggest that *Monotheca buxifolia* possess pain, inflammation and pyrexia ameliorating properties, probably mediated by the presence of oleanolic acid and isoquercetin contents, though the involvement of other important phytochemicals constituents cannot be ignored.

**Electronic supplementary material:**

The online version of this article (doi:10.1186/s12906-016-1257-z) contains supplementary material, which is available to authorized users.

## Background

Discovering excellent remedies for diseases that are efficacious, economical and having minimum adverse effects is the need of the hour. For discovering such products, medicinal plants are considered as best choice, as they provide a wide range of bioactive compounds, making them a rich source of different types of medicines [1]. Majority of drugs currently used in the clinics are due to extensive research on isolation from the natural sources [2].

*Monotheca buxifolia* (Falc.) A. DC. is a member of genus *Monotheca* which belongs to family *Sapotaceae. M. buxifolia* is one of the important tree species of Pakistan that still exhibits dominance in some of the forests, particularly in the Dir District. It is also distributed in the mountains of Afghanistan, northern Oman, and in the south-east Saudi Arabia. *M. buxifolia* is mainly used for fuel, fodder, small timber, roof thatching materials, and notably used as fence around cultivated fields due to its thorny nature. This species also yields fruits, locally called Gurguri, which provides a source of income for the local inhabitants [3]. In folk medicine, *M. buxifolia* fruit is used as hematinic, laxative, purgative, vermicidal, antipyretic, and for the management of gastro-urinary disorders [4–7]. The leaves of *M. buxifolia* contain anthraquinones, flavonoids, terpenoids, cardiac glycosides, saponins, reducing sugars, tannins and poly-phenolic compounds [6]. The total phenolic compounds in *M. buxifolia* fruit fractions, ranged between 59.13 ± 2.6 mg and 16.66 ± 1.3 mg/g dry weight of fraction with the butanolic extract showed the highest total phenolics (59.13 ± 2.6 mg GAE/g fraction). Likewise, the flavonoid contents were in the range of 4.11 ± 0.51 to 48.68 ± 2.8 mg as rutin equivalents/g fraction with highest amount observed in the aqueous fraction (48.68 ± 2.8 mg/g) [8]. Recently, two new compounds, buxifoline-A as alkaloid and buxilide as pyrone were isolated from the ethylacetate fraction of *M. buxifolia* fruit [9]. Flavonoids and poly-phenolic compounds are reported to possess potent anti inflammatory and analgesic properties. Previously, the in vitro antioxidant activity of this fruit has been evaluated and has proved to exhibit potent antioxidant properties [8]. Moreover, *M. buxifolia* fruit also possess inhibitory potential against urease enzyme [9]. The family *Sapotaceae* is widely studied for antimicrobial [10, 11], antioxidant [8], antipyretic [12], CNS depressant [13], anti-inflammatory [14, 15], anthelmintic [16] and antinociceptive activities [12, 17] in various in vitro and in vivo experimental models.

Scientific studies concerning the therapeutic efficacy of *M. buxifolia* are lacking and, in an attempt to provide scientific evidence, we tested the *M. buxifolia* fruit hyrdo-ethanolic (30:70) extract (MBHE) for antinociceptive, anti-inflammatory and antipyretic activities in mice.

## Methods

### Chemicals and drugs

Tramadol (Tramal®, Searle Company (Pvt), Pakistan), diclofenac sodium (Voren®, AsianContinental (Pvt), Pakistan), carrageenan and brewer’s yeast (Sigma-Aldrich).

### Animals

BALB/c mice of either sex (21–35 g) were purchased from the animal house of the Department of Pharmacy, University of Peshawar and were acclimatized at 25 ± 2 °C under a 12 h dark/light cycle for 10 days. Food and water were provided *ad libitum*. The experimental protocols for this study were approved by the Ethical Committee of the Department of Pharmacy, University of Peshawar, Pakistan (registration number: 04/EC-15/Pharm).

### Plant material

Fruits of *Monotheca buxifolia* were collected from the northern areas (District Dir Lower) of Pakistan in August 2013. It was identified by Ghulam Jelani, taxonomist at the Department of Botany, University of Peshawar. Afterwards, a specimen was deposited in the herbarium under the voucher number Bot. 20061 (PUP).

### Preparation of *Monotheca buxifolia* fruit extract

Fruits of *Monotheca buxifolia* (50 kg) were collected and washed to remove dust and other impurities. The seeds were separated and the collected fleshy fruit pulp was dried under shade at ambient temperature. The dried pulp (6.5 kg) was coarsely grinded and was subjected to extraction by adding hydro-ethanolic solvent (40 L) with occasional shaking for 14 days (2 × 7) according to a previous reported method [18]. It was then filtered using a Whatman-1 filter paper. The solvent was evaporated under reduced pressure in a rotary evaporator (BUCHI Rotavapor R-200, Switzerland) at 40 °C until a semisolid mass (2.419 kg) was obtained (yield 4.838 %). The obtained extract was kept in refrigerator till further analysis.

### Phytochemical analysis

MBHE was preliminary evaluated by qualitative phytochemical analysis [19] and was further screened by quantitative analysis of phenolic, flavonoid [20] and triterpenoid [21] contents. MBHE was also subjected to different analytical techniques for isolation and structural elucidation of natural compounds. MBHE (2.304 kg) was mixed with 2.5 L distilled water and soaked overnight, extracted successively with hexanes (3 × 5 L), chloroform (3 × 5 L), ethyl-acetate (3 × 5 L), and *n*-butanol (3 × 5 L) to obtain hexane soluble (56 g), chloroform soluble (57.7 g), ethylacetate soluble (34.9 g) and *n-*butanol soluble (54.5 g) fractions respectively. The ethyl-acetate fraction (30 g) was subjected to vacuum liquid chromatography on normal phase silica gel and eluted using hexane, hexanes-ethylacetate, ethylacetate, ethylacetate-methanol and methanol with increasing polarity to yield 18 fractions (Fr. 1–18). Compound **1** (13 mg) was obtained from fraction 2 (1.2 g) through repeated column chromatography using hexanes-ethylacetate (9:1 – 8:2) while fraction 15 (1.4 g) was re-chromatographed on silica gel column using hexanes-ethylacetate (8:2) to obtained compound **2** (15 mg). Purity of these two compounds was assessed using TLC followed by spraying with ceric sulphate and heating. Their chemical structure was elucidated using ^1^H-NMR, ^13^C-NMR, 2D-NMR, EI-MS, FAB-MS, UV, and IR analytical techniques. The data obtained for these compounds were unambiguously matched with reported data from the literature [22, 23].

### Antinociceptive activity

The antinociceptive activity of MBHE was evaluated by acetic acid induced abdominal constriction assay [24] in BALB/c mice (21–24 g). The animals were withdrawn from food 2 h before the start of experiment. MBHE was administered orally at doses of 50, 100, and 150 mg/kg. Diclofenac sodium was administered at a dose of 50 mg/kg p.o and served as positive control. After 30 min of treatment, all animals were injected i.p with 1 % acetic acid. The number of writhes were counted after 5 min of acetic acid injection and the animals were observed for 20 min. The mean incidence of constrictions in treated groups was compared to untreated controls. Percent protection against nociception was calculated as:

$$ \%\  Protection = \left(1\ \hbox{--}\ Number\  of\  abdominal\  constrictions\  after\  treatment/ Number\  of\  abdominal\  constrictions\  of\  untreated\  control\right) \times 100 $$

### Anti-inflammatory activity

The anti-inflammatory activity of MBHE was tested in the carrageenan induced paw edema assay [25] in BALB/c mice (25–30 g). The animals were starved for 4 h before the start of experiment. MBHE was administered orally at doses of 50, 100, and 150 mg/kg. Aspirin was used as standard and was administered at a dose of 150 mg/kg p.o. After 30 min, all animals were challenged with a 50 μL of 1 % solution of carrageenan, injected subcutaneously into the plantar region of the left hind paw. The paw volume was measured using a digital plethysmometer at 1 to 5 h after challenge with carrageenan. The mean paw swelling across the treated groups was compared to untreated control group. Percent inhibition of paw edema was determined as:

$$ \%\  Inhibition = \left(A\ \hbox{--}\ B\right)/A \times 100 $$

Where A is the paw volume of carrageenan alone treated group and B is the paw volume of tested group.

### Antipyretic activity

The antipyretic potential of MBHE was screened against brewer’s yeast induced pyrexia [26] in BALB/c mice (30–35 g). The animals were deprived of food for 5 h before the start of experiment. Their normal rectal temperature was recorded using a digital thermometer, after which a 15 % solution of brewer’s yeast was injected subcutaneously at a dose of 10 ml/kg to each animal. The rise in body temperature was recorded after 24 h and animals showing at least 0.5 °C rise of their body temperature were included in the experiment. The pyrexic animals were orally administered with MBHE at doses of 50, 100 and 150 mg/kg or acetaminophen, which was used as standard in a dose of 150 mg/kg. The rectal temperature of animals was then recorded at 1 to 5 h duration. The mean elevation of body temperature in °F across treated groups was compared to that of untreated control.

### Statistical analysis

Data were expressed as mean ± S.E.M or SD. Statistical analysis was performed by one way ANOVA followed by Dunnett’s or Tukey’s *post hoc* test where appropriate using GraphPad Prism 5 (GraphPad Software Inc. San Diego CA, USA). Statistical significance was deduced at *P* ≤ 0.05.

## Results

### Phytochemical analysis of *Monotheca buxifolia*

**Fig. 1:**
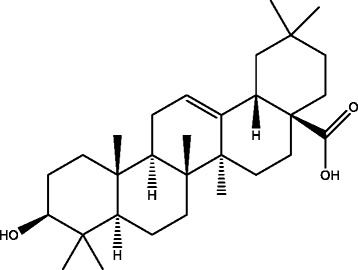
Structure of compound **1** (Oleanolic acid)

**Fig. 2:**
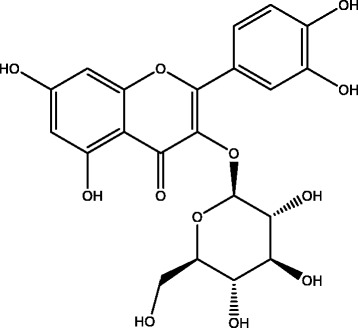
Structure of compound **2** (Isoquercetin)

Preliminary qualitative analysis of MBHE disclosed the presence of alkaloids, phenolics, flavonoids, athraquinones glycosides, saponins, and triterpenoids (Table 1), while quantitative analysis revealed (mean ± SEM, *n* = 5) the presence of phenolics (49.60 ± 1.93 as mg gallic acid equivalent/g dry weight), flavonoids (44.80 ± 1.65 as mg rutin equivalent/g dry weight) and triterpenoids (32.20 ± 2.05 as mg ursolic acid equivalent/g dry weight). Subsequent more detailed isolation and structural determination techniques led to the isolation of compound **1** as oleanolic acid (Fig. 1) and compound **2** as isoquercetin (Fig. 2) from MBHE. Oleanolic acid showed molecular ion peak at m/z at 456, and having a molecular formula of C_30_H_48_O_3_ at m/z 456.7003 (Calc. 456.6840) (Additional file 1: Figure S1–S6). Isoquercetin showed a pseudo-molecular ion peak [M-H] at *m/z* 463 and having a molecular formula of C_21_H_20_O_12_ at *m/z* 463.0012 (Additional file 1: Figure S7-S16).

**Table 1:** Qualitative phytochemical analysis of *Monotheca buxifolia*

Phytochemical class	Protocol	Result
Alkaloids	Mayer’s test	+
	Dragendorff’s test	+
	Wagner’s test	+
Phenolics	Ferric chloride test	+
Flavonoids	Lead acetate test	+
	Ethyl acetate test	+
	Sodium hydroxide test	+
Anthraquinone glycosides	Borntrager’s test	+
Saponins	Foam test	+
Triterpenoids	Liebermann-Burchard test	+

### *Monotheca buxifolia* attenuates tonic visceral chemical induced nociception

**Fig. 3:**
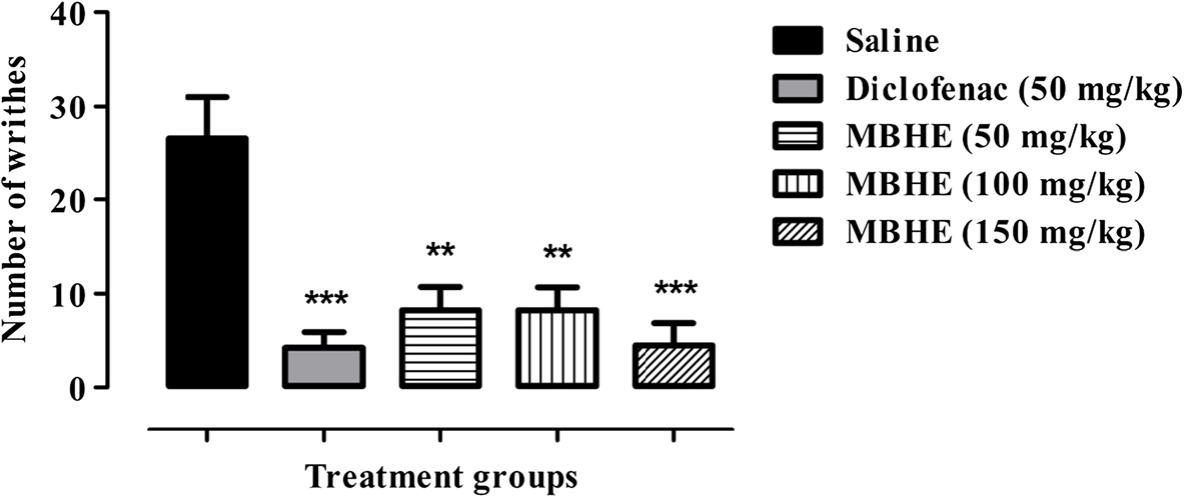
Antinociceptive activity of *Monotheca buxifolia* hydro-ethanolic extract (MBHE) in acetic acid induced abdominal constriction assay. Values expressed as mean number of writhes ± SEM. ANOVA followed by Dunnett’s *post hoc* test. *P < 0.05, **P < 0.01, ***P < 0.001 compared to saline treated group. *n* = 6 animals per group

As shown in Fig. 3, significant attenuation (*P* < 0.01) of acetic acid induced visceral pain was demonstrated by MBHE at doses of 50 mg/kg (68.87 %) and 100 mg/kg (68.87 %). However, a highly significant (*P* < 0.001) antinociceptive effect was observed at 150 mg/kg (83.02 %). Similarly, the standard diclofenac sodium at 50 mg/kg produced significant amelioration (*P* < 0.001, 83.97 %) of acetic acid induced writhes.

### *Monotheca buxifolia* alleviates carrageenan induced paw edema

As shown in Table 2, carrageenan alone treated animals exhibited significant (*P* < 0.001) paw edema after 1 h of administration and this edema remained persistent for 5 h. Pretreatment with MBHE significantly alleviated the carrageenan elicited paw edema after 1 and 2 h at doses of 50 mg/kg (*P* < 0.01, 22.94 and 23.78 %), 100 mg/kg (*P* < 0.001 and *P* < 0.01, 33.23 and 25.13 %) and 150 mg/kg (*P* < 0.001, 38.23 and 30.54 %). After 3 and 4 h, high significant reduction (*P* < 0.001) of paw edema was produced by all the tested doses i.e. 50 mg/kg (27.34 and 40.41 %), 100 mg/kg (26.58 and 41.43 %) and 150 mg/kg (27.34 and 44.49 %), however; a less significant reduction (*P* < 0.01) was observed with these doses after 5 h (20, 21.13 and 25 %) of carrageenan administration. Moreover, the standard aspirin at a dose of 150 mg/kg produced moderate to high significant alleviation of paw edema after 1 h (*P* < 0.01, 25 %) and 2–5 h (*P* < 0.001, 32.43, 36.20, 33.67 and 30.68 %) duration of challenge with carrageenan.

**Table 2:** Anti-inflammatory activity of *Monotheca buxifolia* hydro-ethanolic extract (MBHE) in carrageenan induced paw edema in mice

Treatment	1st h	2nd h	3rd h	4th h	5th h
Group 1 Saline	0.235 ± 0.012	0.227 ± 0.026	0.240 ± 0.011	0.210 ± 0.016	0.230 ± 0.036
Group 2 Carrageenan	0.340 ± 0.016^###^	0.370 ± 0.028^###^	0.395 ± 0.025^###^	0.490 ± 0.057^###^	0.440 ± 0.028^###^
Group 3 Aspirin (150 mg/kg)	0.255 ± 0.028**	0.250 ± 0.035***	0.252 ± 0.020***	0.325 ± 0.052***	0.305 ± 0.019***
Group 4 (MBHE 50 mg/kg)	0.262 ± 0.022**	0.282 ± 0.012**	0.287 ± 0.025***	0.292 ± 0.015***	0.352 ± 0.029**
Group 5 (MBHE 100 mg/kg)	0.227 ± 0.040***	0.277 ± 0.035**	0.290 ± 0.046***	0.287 ± 0.037***	0.347 ± 0.029**
Group 6 (MBHE 150 mg/kg)	0.210 ± 0.028***	0.257 ± 0.026***	0.287 ± 0.022***	0.272 ± 0.044***	0.330 ± 0.038**

Values are expressed as mean paw volume in ml ± SD. ANOVA followed by Tukey’s *post hoc* test. ^###^ *P* < 0.001 compared to group 1. **P < 0.01, ***P < 0.001 compared to group 2. *n* = 6 animals per group

### *Monotheca buxifolia* relieves brewer’s yeast induced pyrexia

As shown in Table 3, brewer’s yeast induced significant increase (*P* < 0.001) of body temperature which remained elevated throughout the 5 h study period. After 1 h of treatment, significant reduction (*P* < 0.001) in pyrexia was noticed with MBHE at doses of 100 and 150 mg/kg. Almost, similar protective effect was observed after 2 h of treatment with MBHE at 100 mg/kg (*P* < 0.01) and 150 mg/kg (*P* < 0.001). After 3 h, less significant antipyretic effect was observed with the 100 mg/kg (*P* < 0.05) and 150 mg/kg (*P* < 0.01) doses of MBHE and this effect persisted for the 5 h. The 50 mg/kg dose of MBHE was only effective at 2nd hour, at which a less significant reduction (*P* < 0.05) of pyrexic response was observed. The standard acetaminophen at 150 mg/kg produced favorable antipyretic effect (*P* < 0.001) throughout the study period (5 h).

**Table 3:** Antipyretic activity of *Monotheca buxifolia* hydro-ethanolic extract (MBHE) in brewer’s yeast induced pyrexia in mice

Treatment	1st h	2nd h	3rd h	4th h	5th h
Group 1 (Brewer’s yeast + Saline)	100.4 ± 0.358	100.5 ± 0.346	100.0 ± 0.335	100.4 ± 0.265	100.5 ± 0.341
Group 2 (Acetaminophen 150 mg/kg)	96.18 ± 0.757^***^	96.62 ± 0.755^***^	95.50 ± 1.023^***^	96.07 ± 0.720^***^	95.23 ± 0.731^***^
Group 3 (MBHE 50 mg/kg)	97.93 ± 0.449	98.20 ± 0.634^*^	98.58 ± 0.838	99.27 ± 0.860	99.88 ± 0.812
Group 4 (MBHE 100 mg/kg)	96.02 ± 0.884^***^	97.18 ± 0.570^**^	97.68 ± 0.244^*^	97.72 ± 0.342^*^	97.42 ± 0.709^*^
Group 5 (MBHE 150 mg/kg)	93.70 ± 0.753^***^	96.93 ± 0.584^***^	96.48 ± 0.303^**^	97.33 ± 0.547^**^	96.35 ± 0.837^**^

Values are expressed as mean rectal temperature in °F ± SD. ANOVA followed by Dunnett’s *post hoc* test. *P < 0.05, **P < 0.01, ***P < 0.001 compared to group 1. *n* = 6 animals per group

## Discussion

Fruit of *Monitica buxifollia* was screened for its potential antinociceptive, anti-inflammatory, and antipyretic activities. The nociceptive response in the acetic acid induced writhing test results from the production of prostaglandins through the action of cyclooxygenases. The liberated prostaglandins stimulate sensory pathways in the mouse peritoneum and incite viscero-somatic y reflexes manifested as strong abdominal constrictions or writhes. The acetic acid induced writhes are sensitive to various analgesics. MBHE when administered at doses of 50, 100 and 150 mg/kg depressed the acetic acid induced writhes and the antinociceptive effect was analogous to that of the standard diclofenac sodium (50 mg/kg).

Carrageenan-induced mice paw edema assay is a widely used test to determine the anti-inflammatory activity of both natural and synthetic compounds. Edema formation due to carrageenan administration in mouse paw is a biphasic event. The initial phase lasting about 1–5 h is predominately characterized by a non-phagocytic edema and has been attributed to the action of various mediators including histamine, serotonin and bradykinin on vascular permeability [27]. The initial phase is followed by a second phase having a duration of 2–5 h and results from overproduction of prostaglandins [28]. It has been reported that the second phase of edema is sensitive to drugs like hydrocortisone, phenylbutazone and indomethacin. In the present study, we have observed potential anti-inflammatory activity of MBHE at all the tested doses (50, 100 and 150 mg/kg) and was comparable to the classical cyclooxygenase inhibitor, aspirin (150 mg/kg).

Brewer’s yeast is an exogenous pyrogen which produces pathogenic fever by binding to lipopolysaccharide binding protein and results in the release of different cytokines like interleukin 1, 6, tumor necrosis factor alpha and prostaglandins. These pro-inflammatory mediators cross the blood brain barrier and act on hypothalamus causing the release of prostaglandin E_2_ which is produced through the action of cyclo-oxygenase-2 and thus increases the body temperature [29]. In this study, potential antipyretic effect was observed for the 100 and 150 mg/kg doses of MBHE and the effect was similar to that of acetaminophen (150 mg/kg).

Biologically active molecules derived from herbal extracts can be used to treat acute and chronic diseases [30]. In the current study, the chromatographically isolated compounds i.e. isoquercetin have been reported to possess neuroprotective [31], cardioprotective [32], hepatoprotective [33], nephroprotective [34], chemopreventive [35], analgesic [36], antioxidant [37] and anti-inflammatory [38] properties. In addition, oleanolic acid has anti-tumor [39], anti-inflammatory [40], antioxidant [41], anti-hyperlipidemic [42], anti-ulcer [43], anti-microbial [44] and analgesic [45, 46] activities. Keeping in view, the potent antinociceptive, anti-inflammatory, and antipyretic activity of MBHE, it can be argued that these beneficial effects can be attributed at least to the presence of isoquercetin and oleanolic acid contents in *M. buxifolia*. However, the involvement of other phytochemical constituents cannot be ignored in the mediation of pain, inflammation and pyrexia relieving properties as *M. buxifolia* has been reported to contain high amount of phenolics and flavonoids [8], β-sitosterol [47], tannins, tri-terpenoids, glycosides, anthraquinones and alkaloids [48] which is further corroborated in this study. Flavonoids are polyphenolic compounds that occur ubiquitously in plants and having a variety of biological effects including antioxidant, anti-inflammatory, antihypertensive, anti-atherosclerotic, anti-tumor, anti-thrombogenic, anti-osteoporotic, antimicrobial, antiviral, anti-ulcerogenic, and antihepatotoxic activities [49, 50]. Naturally occurring anthraquinones are a group of secondary metabolites that possess anti-inflammatory, anti-cancer, antidiabetic, antimicrobial and laxative properties [51]. Similarly, other phytochemical constituents like glycosides and alkaloids also have known beneficial pharmacological effects both in vitro and in vivo [52, 53].

## Conclusion

*Monotheca buxifolia* hydroethanolic extract attenuated the tonic visceral chemical induced nociception and alleviated a phlogistic agent induced inflammatory response. It also relieved a pyrogenic substance induced elevation of body temperature. Phytochemical analysis revealed that *M. buxifolia* contained isoquercetin and oleanolic acid that might be involved in allay of pain, inflammation and pyrexia. Though, the involvement of other phytochemical constituents cannot be ignored in mediating the antinociceptive, anti-inflammatory and antipyretic effects, as *M. buxifolia* also have sufficient amount of flavonoids, anthraquinones glycosides, triterpenoids, and alkaloids. Our findings suggest that *M. buxifolia* is able to mend disease conditions afflicted with pain, inflammation and pyrexia. Further studies are warranted like HPLC-DAD profile to elucidate the exact chemical constituents responsible for these beneficial pharmacological effects.

## Additional file

Additional file 1: Supporting material. (DOCX 12960 kb)
