# Supplementary material for: Pharmacological screening of Monotheca buxifolia (Falc.) A. DC. for antinociceptive, anti-inflammatory and antipyretic activities
Source: BMC Complement Altern Med. 2016 Aug 5;16:273. doi: 10.1186/s12906-016-1257-z (PMC4974707; doi:10.1186/s12906-016-1257-z)
Supplement: Additional file 1: — Supporting material. (DOCX 12960 kb) [file 12906_2016_1257_MOESM1_ESM.docx]

**SUPPORTING MATERIAL**

**Pharmacological screening of *Monotheca buxifolia* (Falc.) A. DC. for antinociceptive, anti-inflammatory and antipyretic activities**

Irfan Ullah^1,2^, Jamshaid Ali Khan^1^, Muhammad Shahid^1^, Ajmal Khan^3^, Achyut Adhikari^4^,

Peer Abdul Hannan^1^, Ibrahim Javed^5^, Faisal Shakeel^1^, Umar Farooq^3^

**Affiliations**

1. Department of Pharmacy, University of Peshawar, Peshawar, Pakistan
2. Department of Pharmacy, Abasyn University, Peshawar, Pakistan
3. Department of Chemistry, COMSATS Institute of Information Technology, Abbottabad, Pakistan.
4. HEJ Research Institute of Chemistry, International Centre for Chemical and Biological Sciences, University of Karachi, Karachi, Pakistan
5. Department of Chemistry, SBA School of Science and Engineering, Lahore University of Management Sciences, Lahore, Pakistan.

**Email Addresses**

Irfan Ullah ([irfanullah@upesh.edu.pk](mailto:irfanullah@upesh.edu.pk))

Jamshaid Ali Khan ([jamshaidkhan@upesh.edu.pk](mailto:jamshaidkhan@upesh.edu.pk))

Muhammad Shahid ([shahidsalim_2002@hotmail.com](mailto:shahidsalim_2002@hotmail.com))

Ajmal Khan (ajmalchemist@yahoo.com

Achyut Adhikari ([adhikarimine@yahoo.com](mailto:adhikarimine@yahoo.com))

Peer Abdul Hannan (peer_hannan@yahoo.com)

Ibrahim Javed (ibrahim.javed@lums.edu.pk)

Faisal Shakeel (faisalshakeel1@gmail.com)

Umar Farooq (umarf@ciit.net.pk)

**Corresponding Author**

Professor Dr. Jamshaid Ali Khan

Department of Pharmacy

University of Peshawar

Peshawar, 25120

Khyber Pakhtunkhwa,

Pakistan

Email: [jamshaidkhan@upesh.edu.pk](mailto:jamshaidkhan@upesh.edu.pk)

Phone: +92919216750

**Phytochemical analysis of Compound 1 (Oleanolic acid)**

**
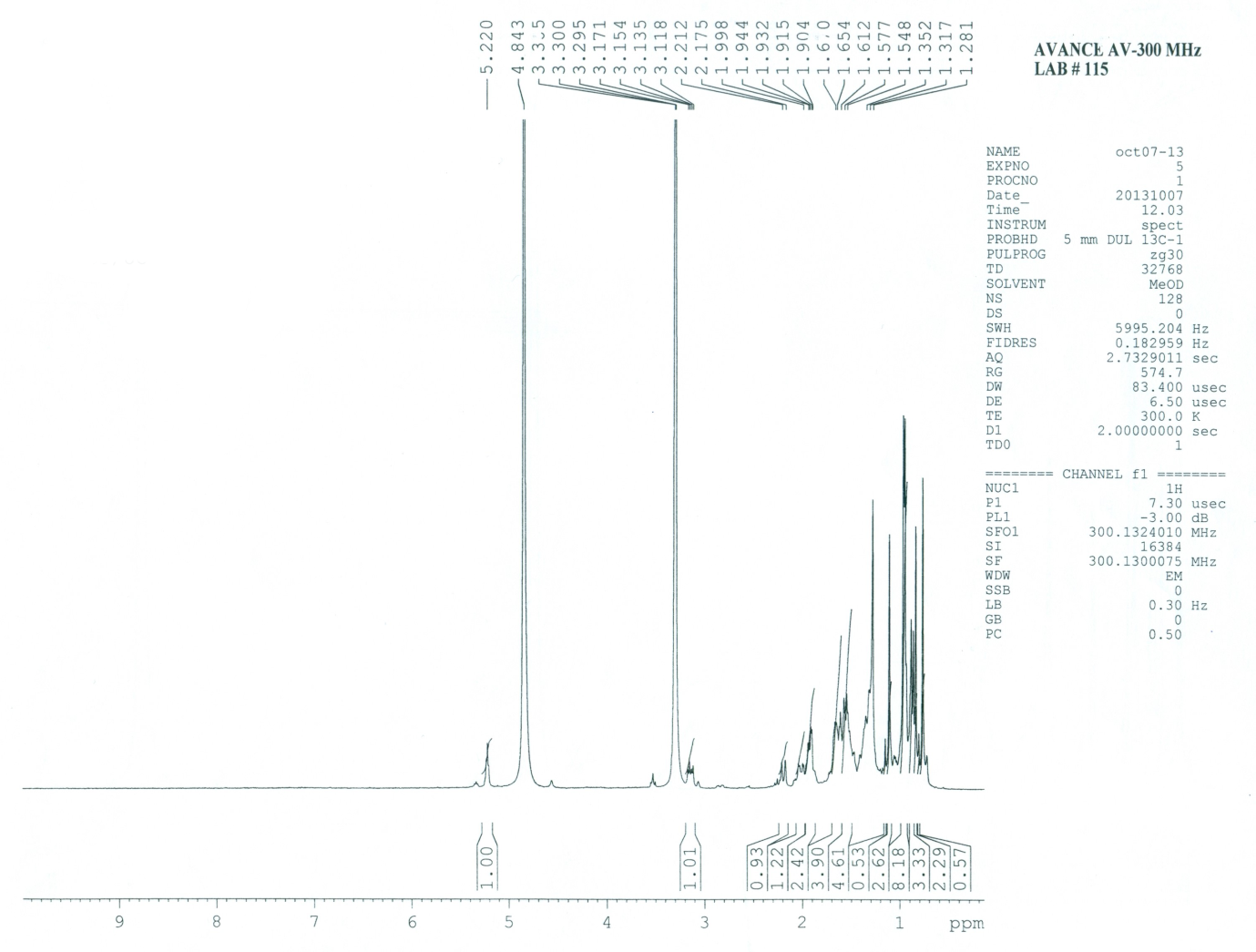
**

**Figure S1:** ^1^H-NMR

**
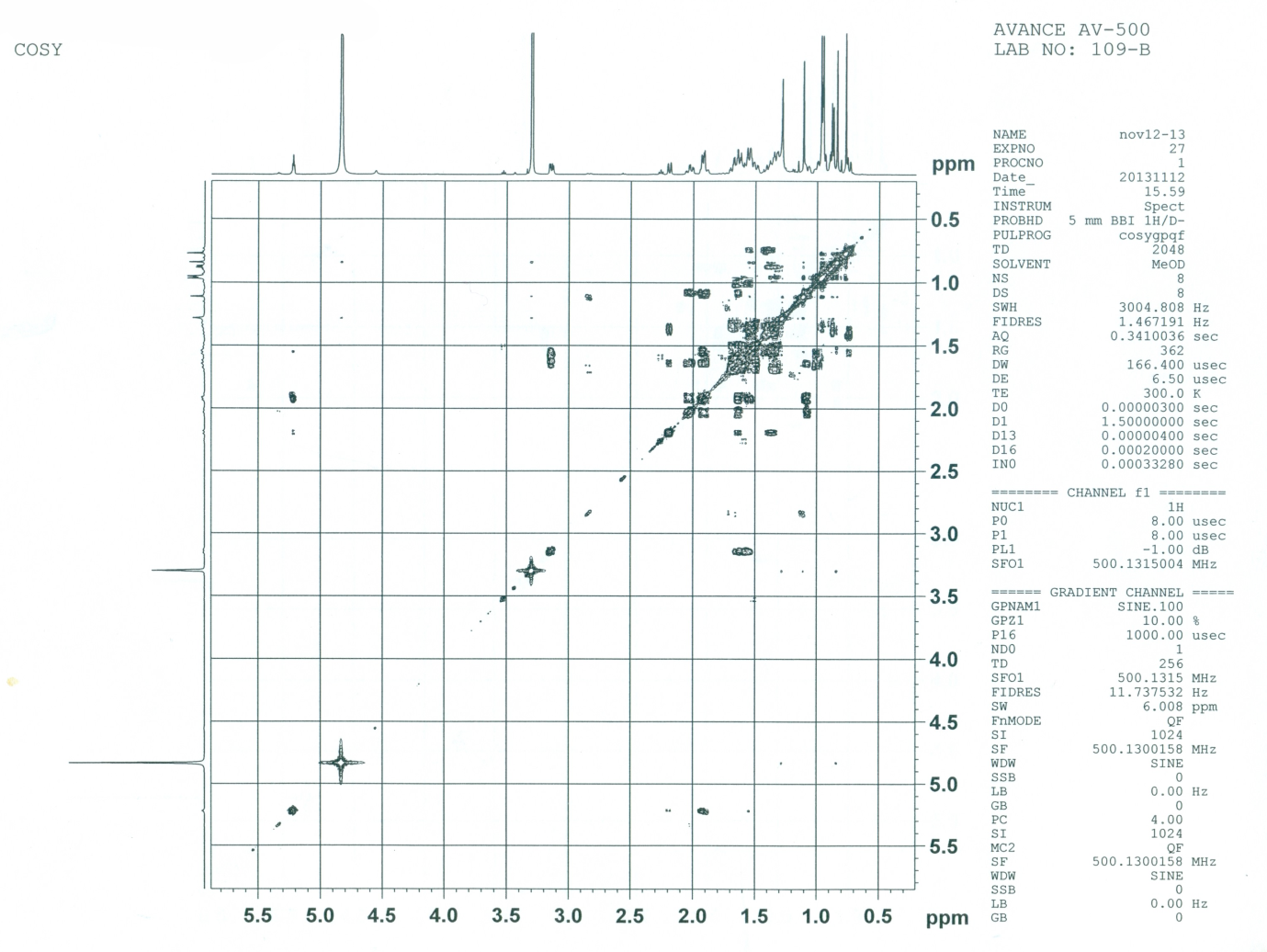
**

**Figure S2:** gCOSY spectrum

**
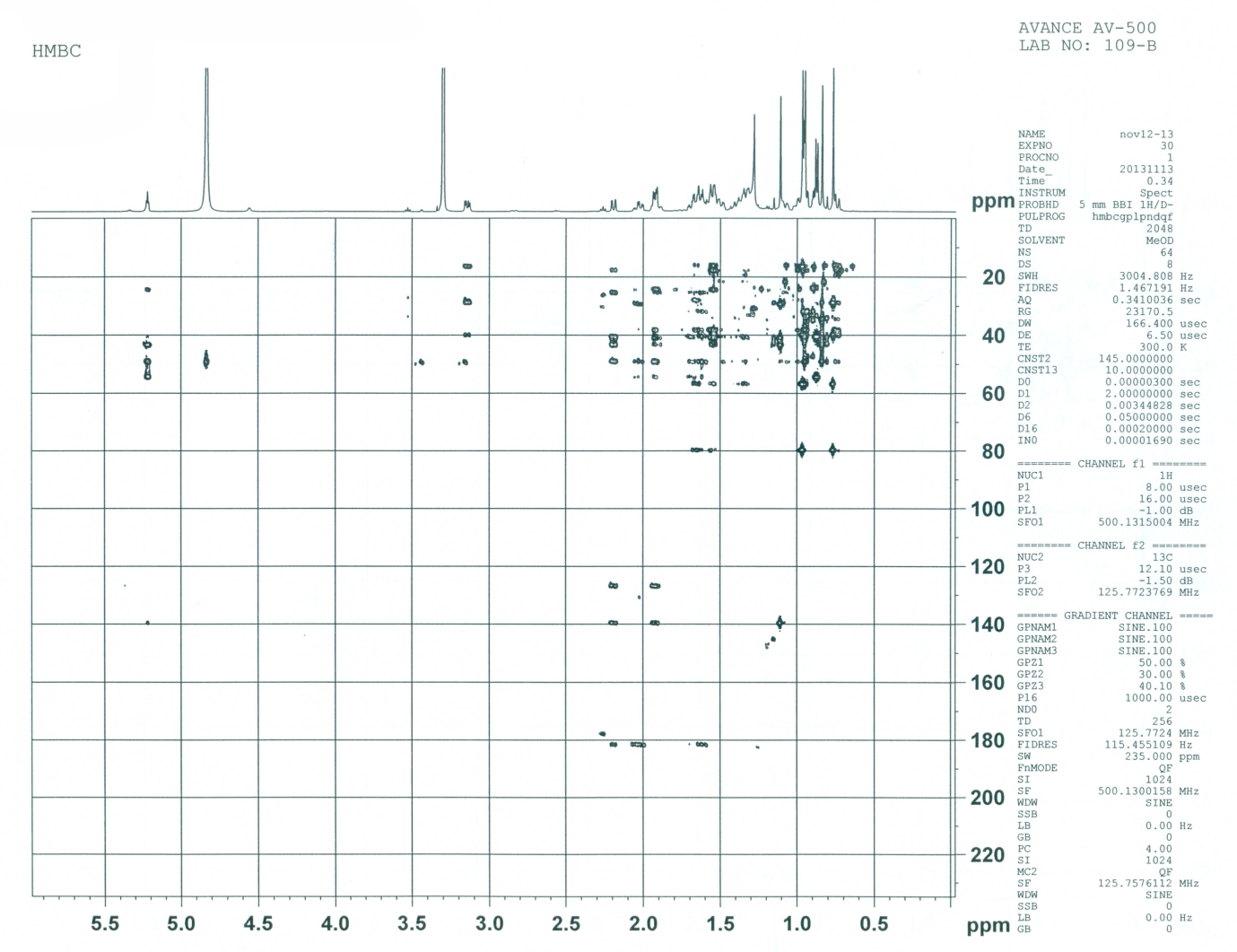
**

**Figure S3:** gHMBC spectrum

**
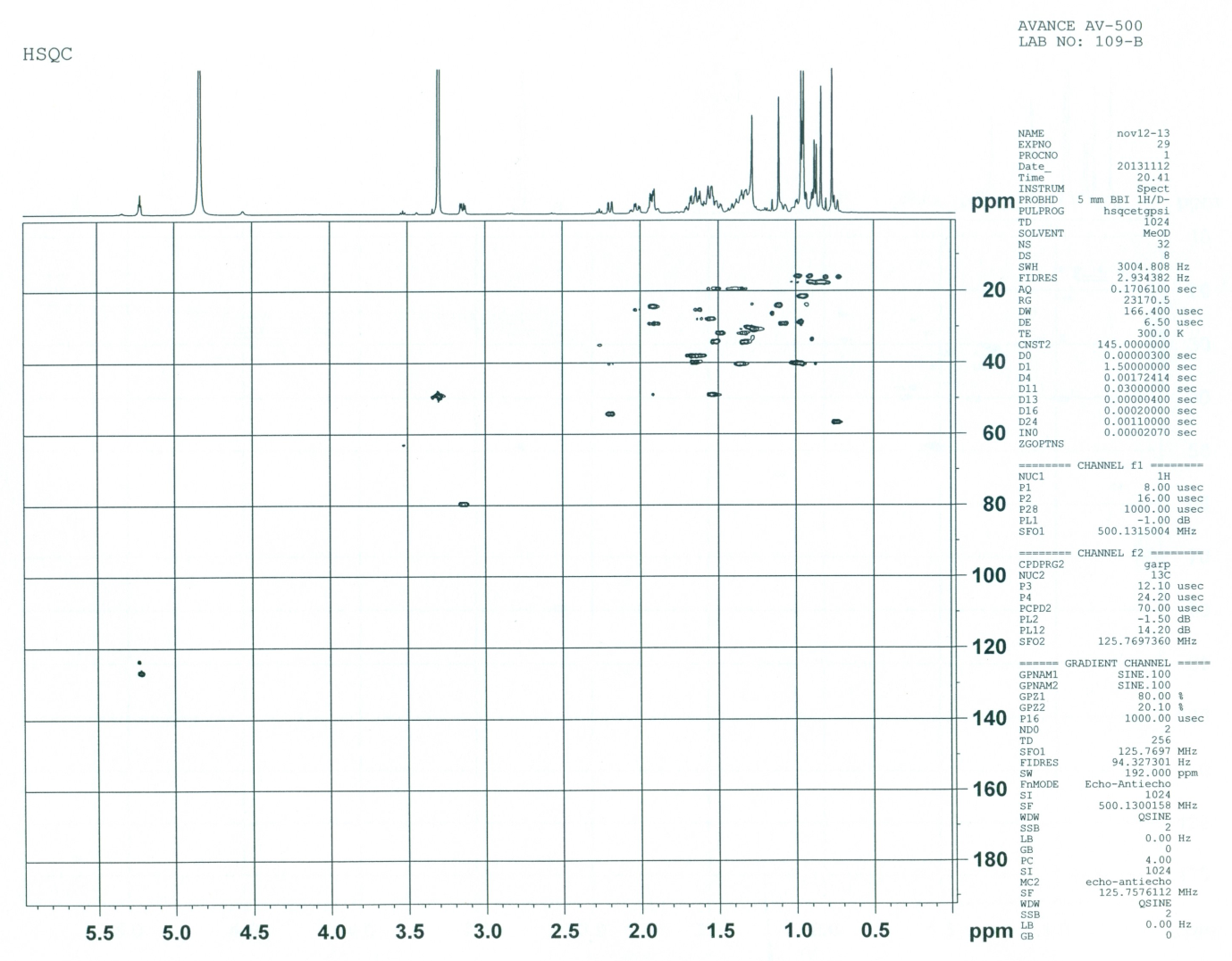
**

**Figure S4:** gHSQC spectrum

**
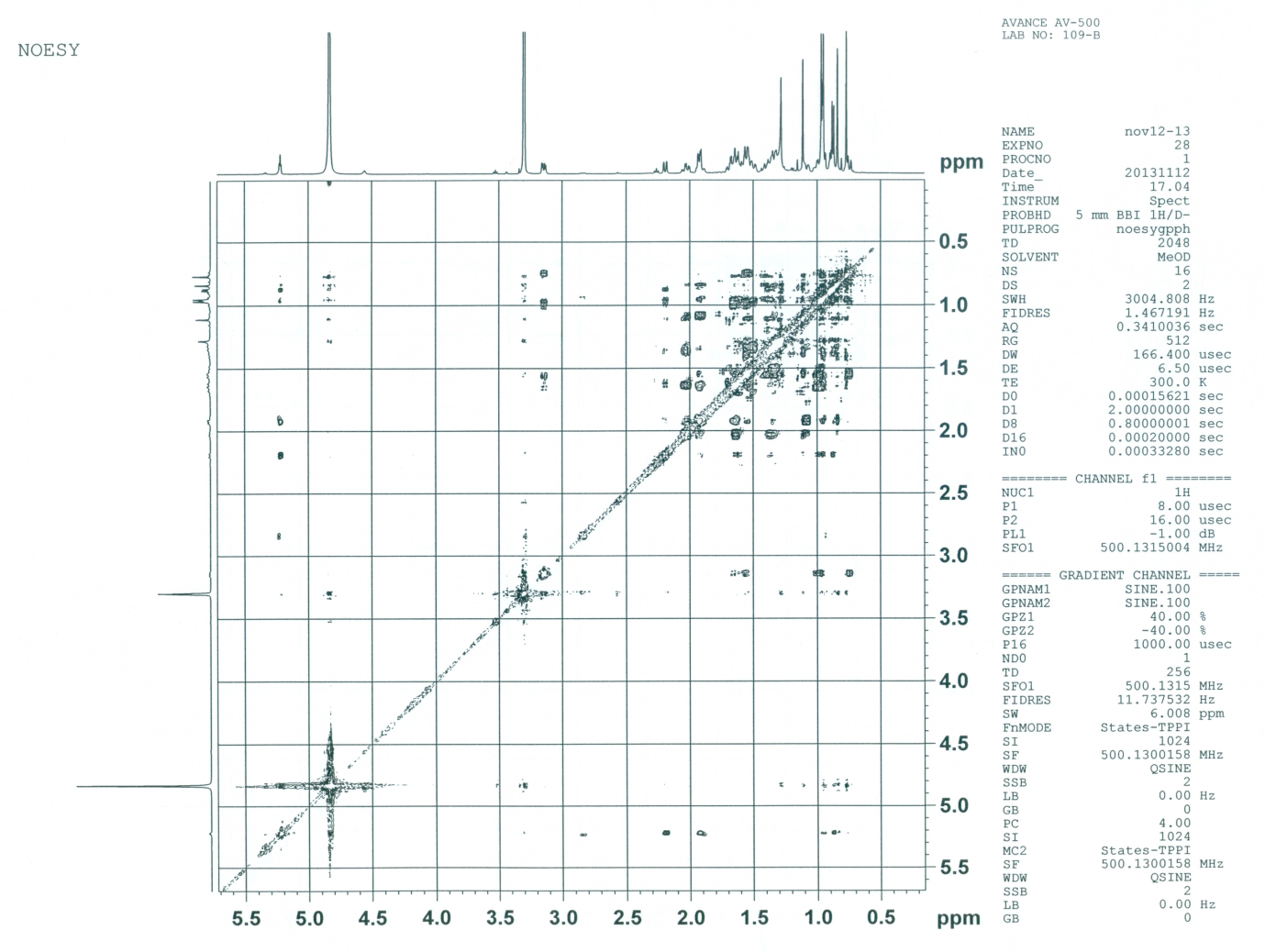
**

**Figure S5:** NOESY spectrum

**
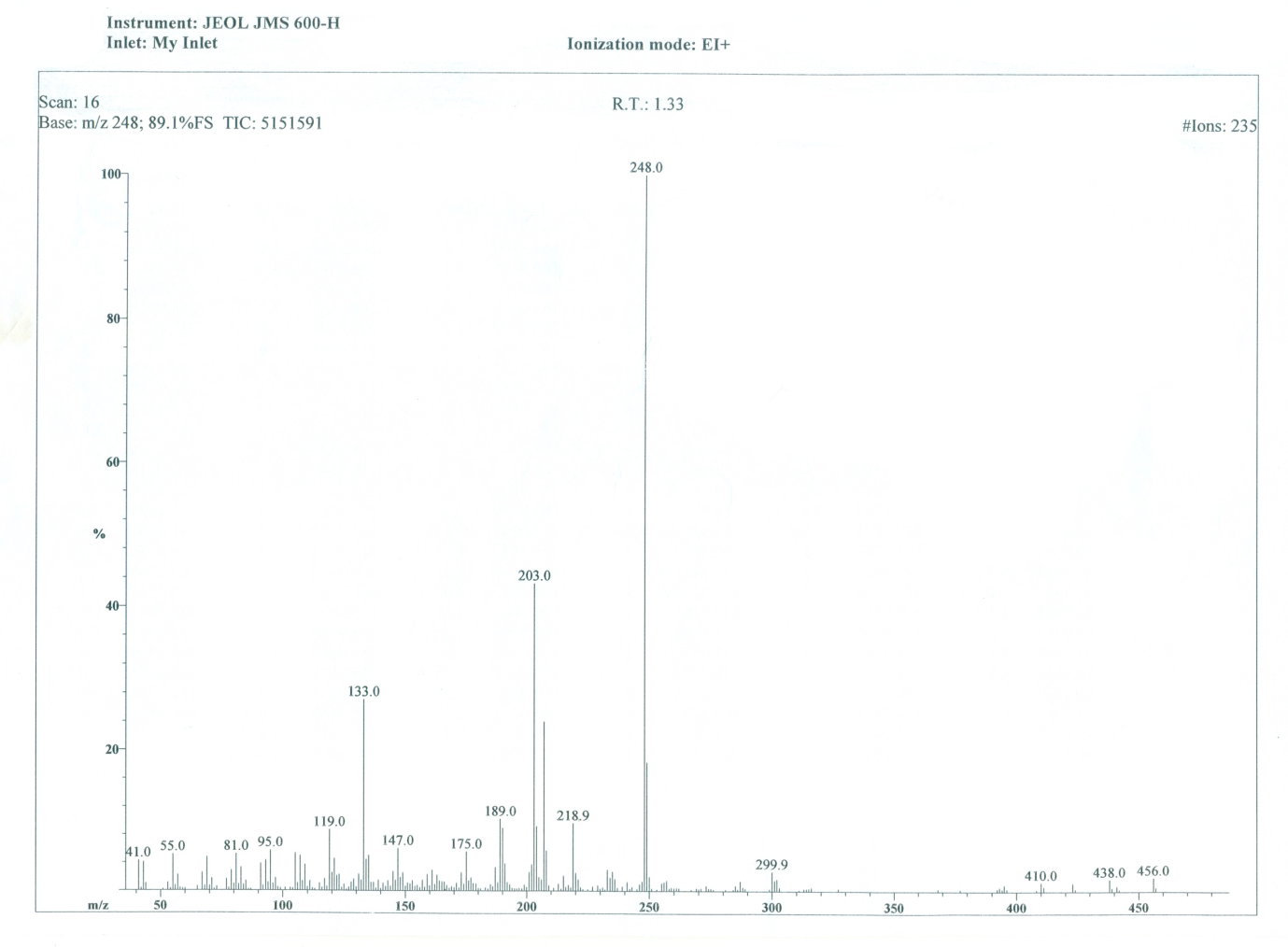
**

**Figure S6:** Mass spectrum

**Phytochemical analysis of Compound 2 (Isoquercetin):**

**
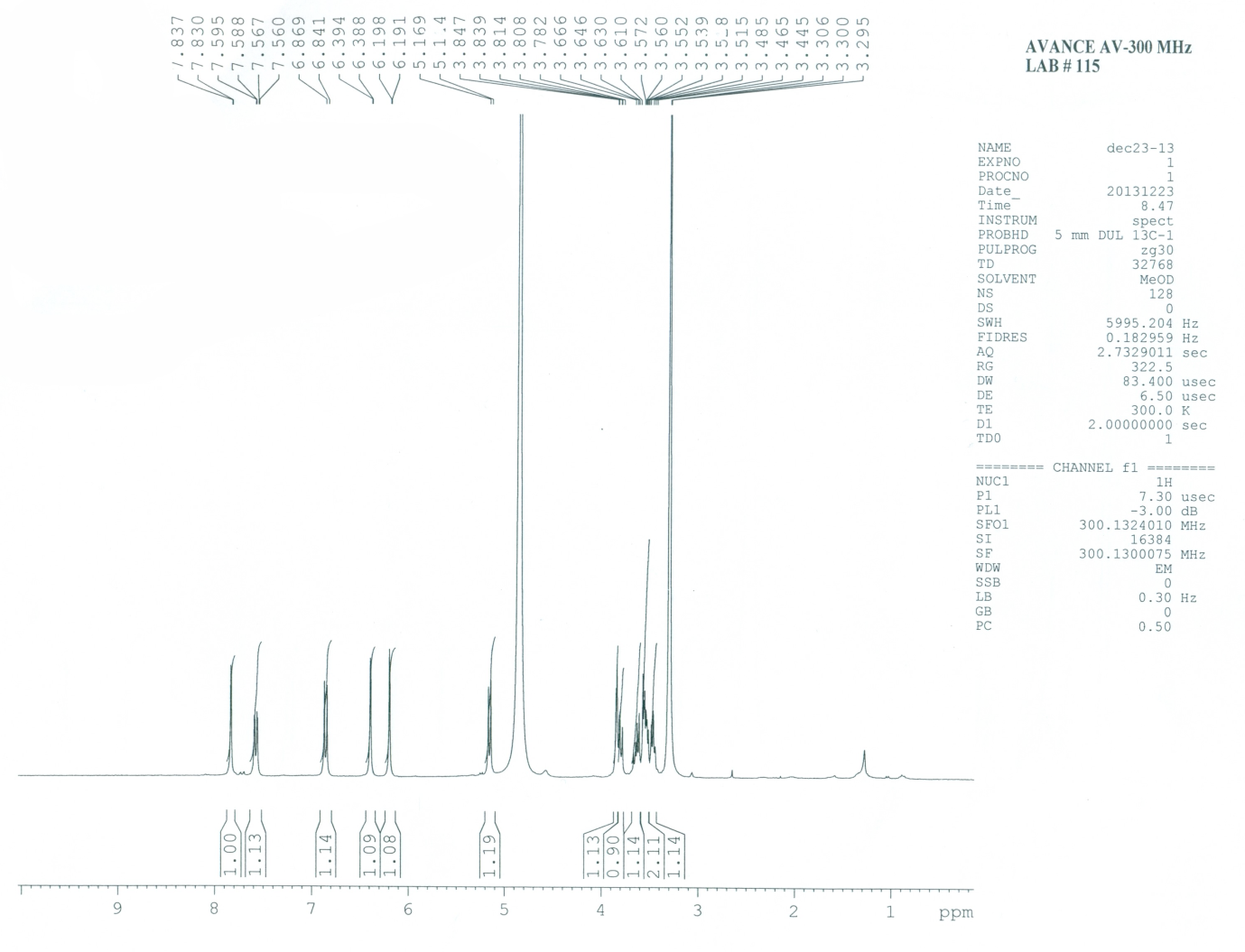
**

**Figure S7:** ^1^H-NMR

**
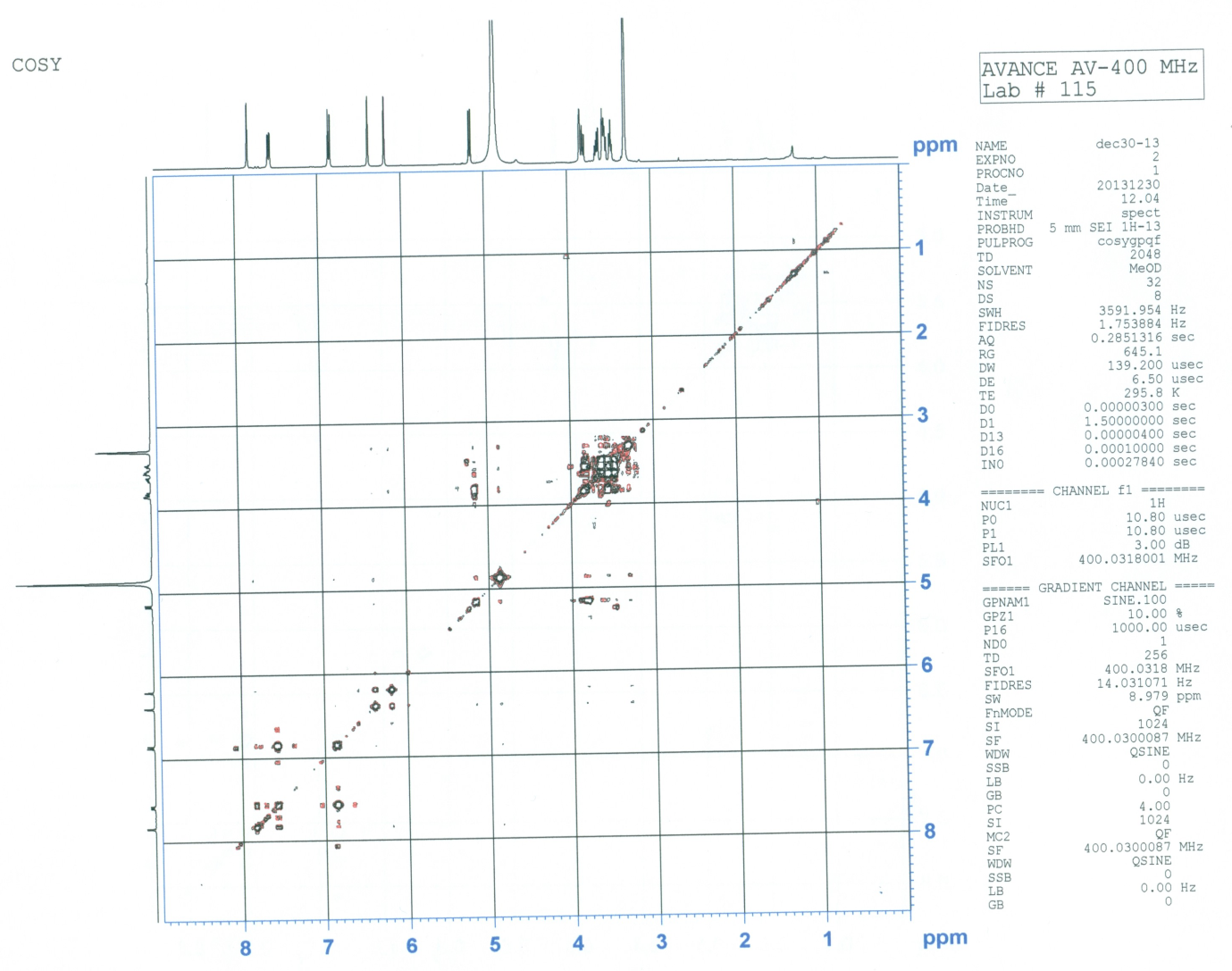
**

**Figure S8:** gCOSY spectrum

**
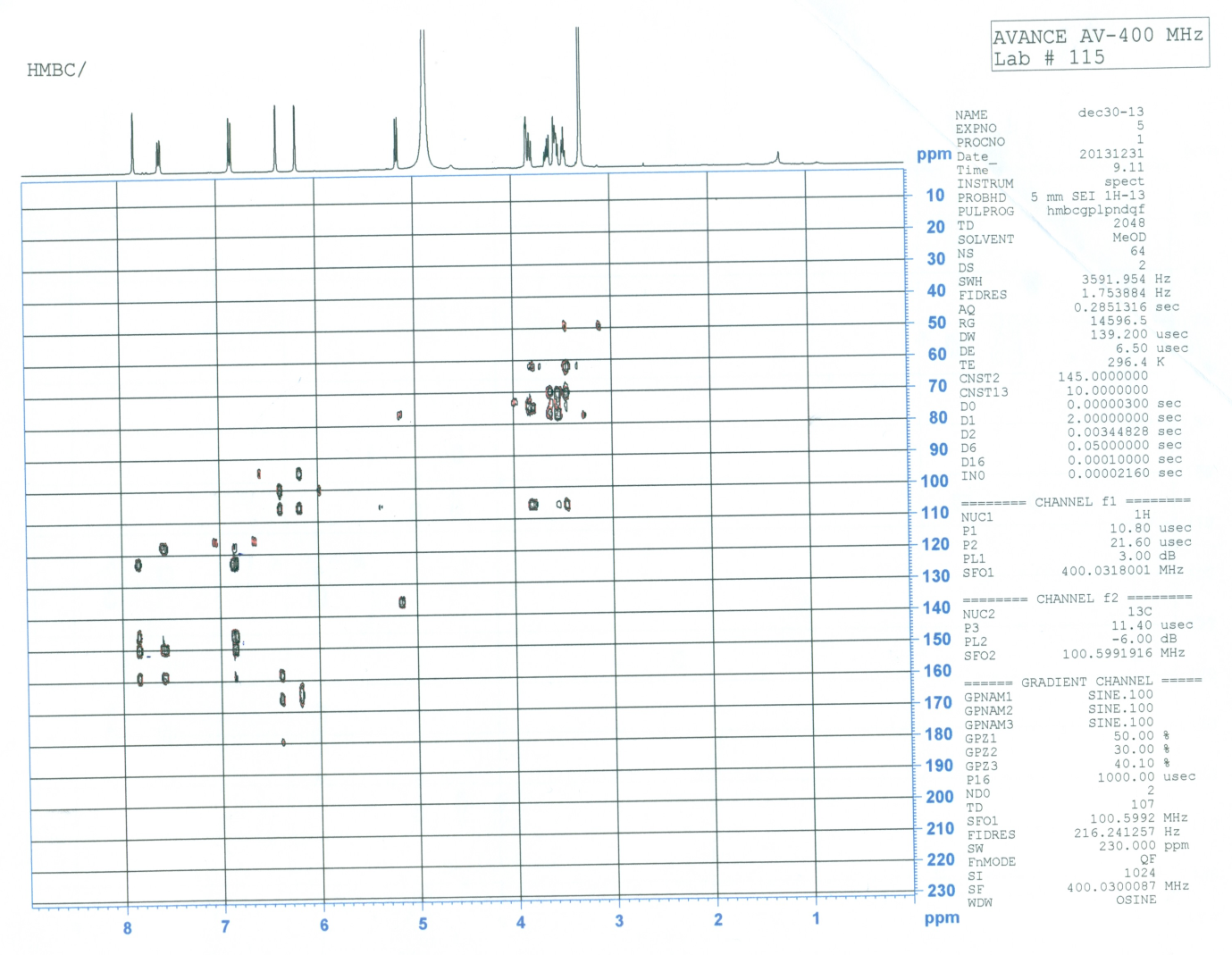
**

**Figure S9:** gHMBC spectrum

**
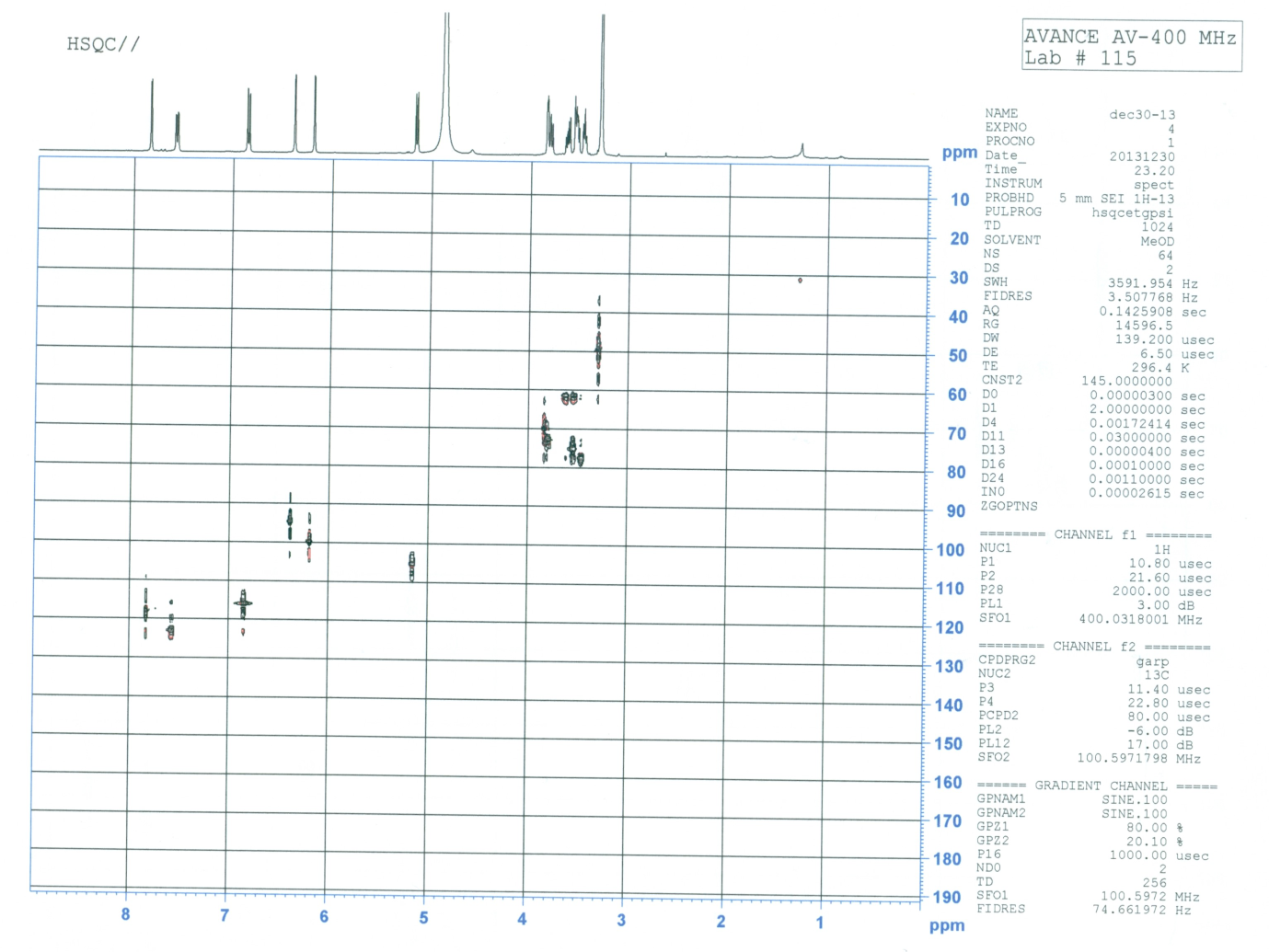
**

**Figure S10:** gHSQC spectrum

**
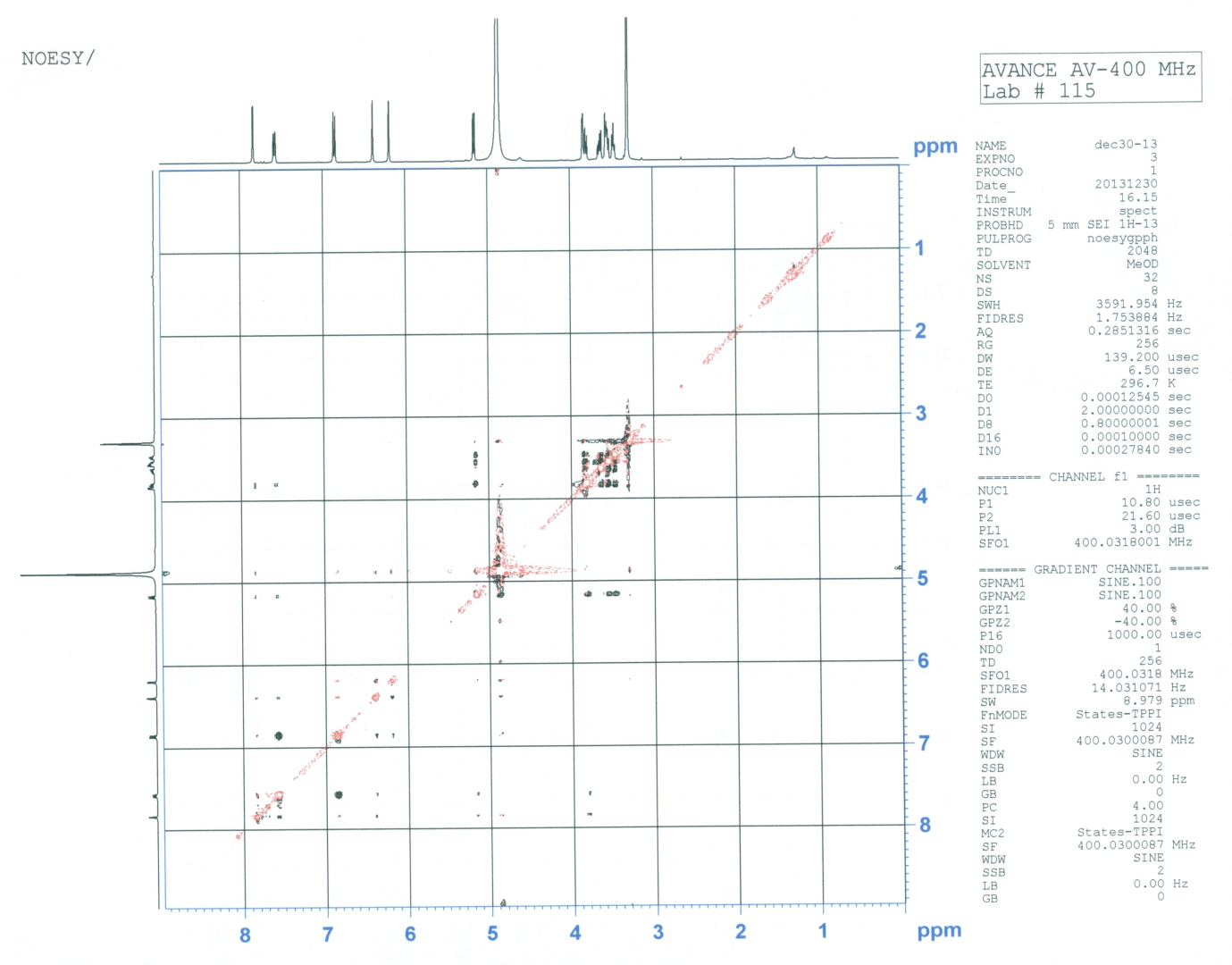
**

**Figure S11:** NOESY spectrum

**
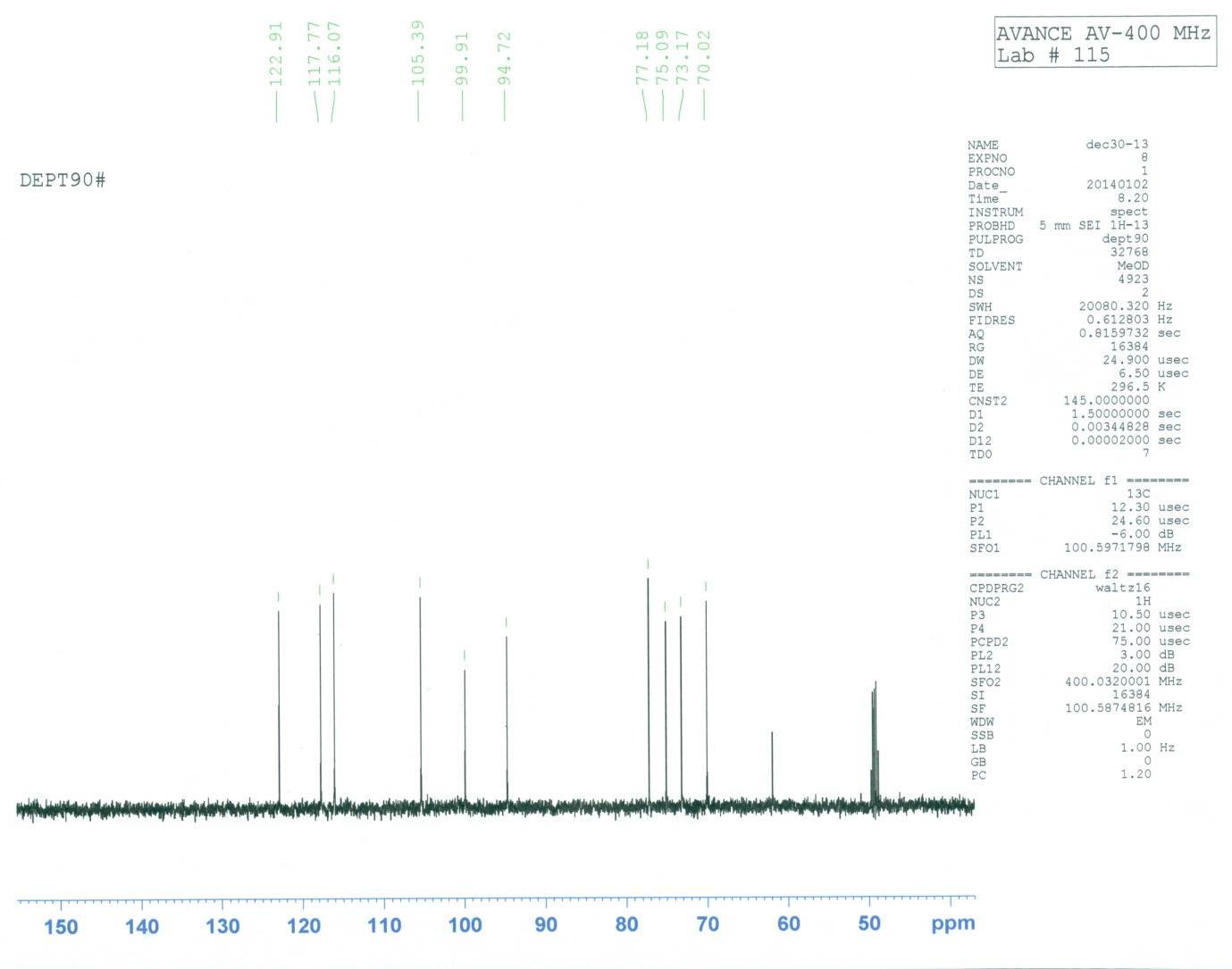
**

**Figure S12:** DEPT 90 spectrum

**
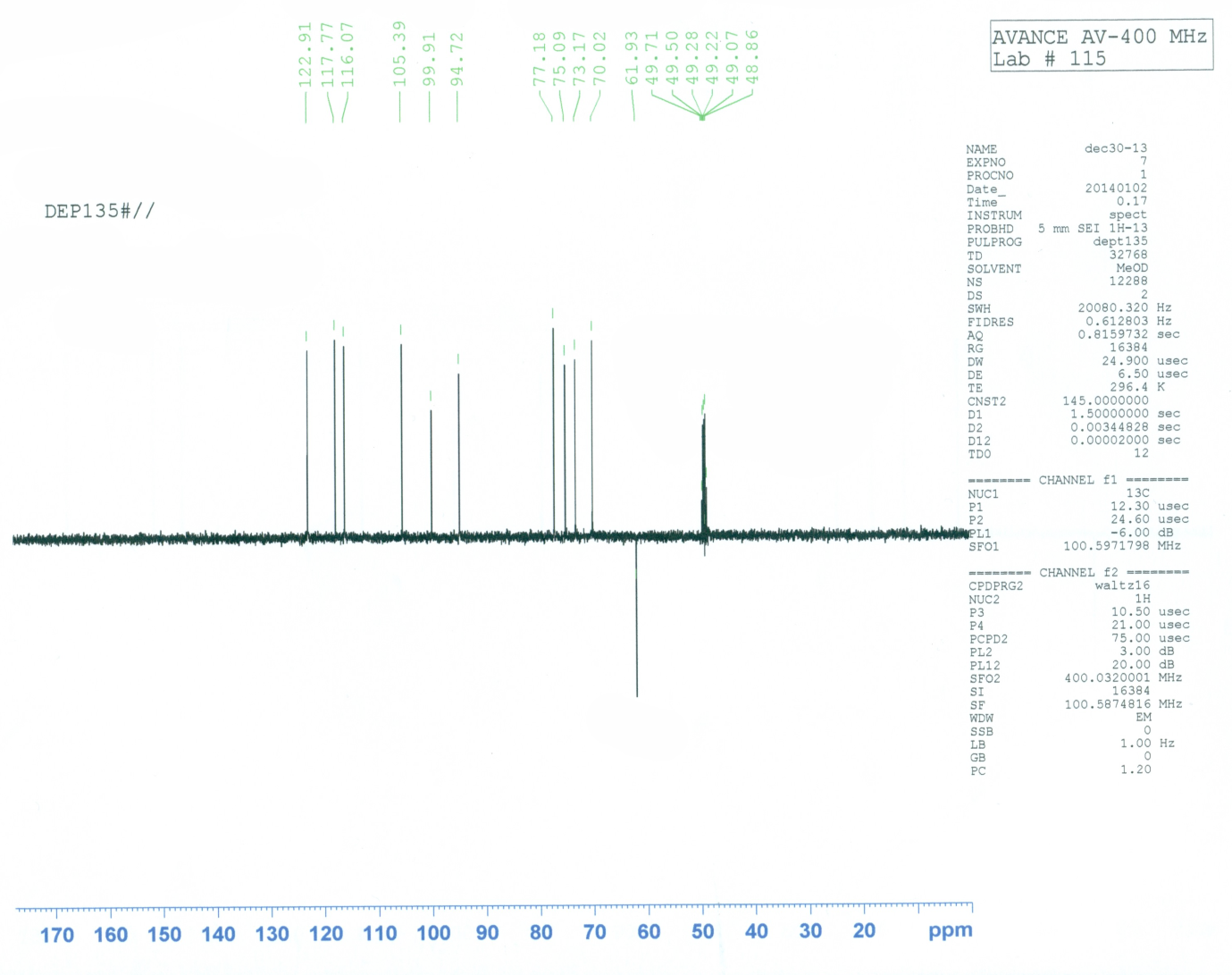
**

**Figure S13:** DEPT 135 spectrum

**
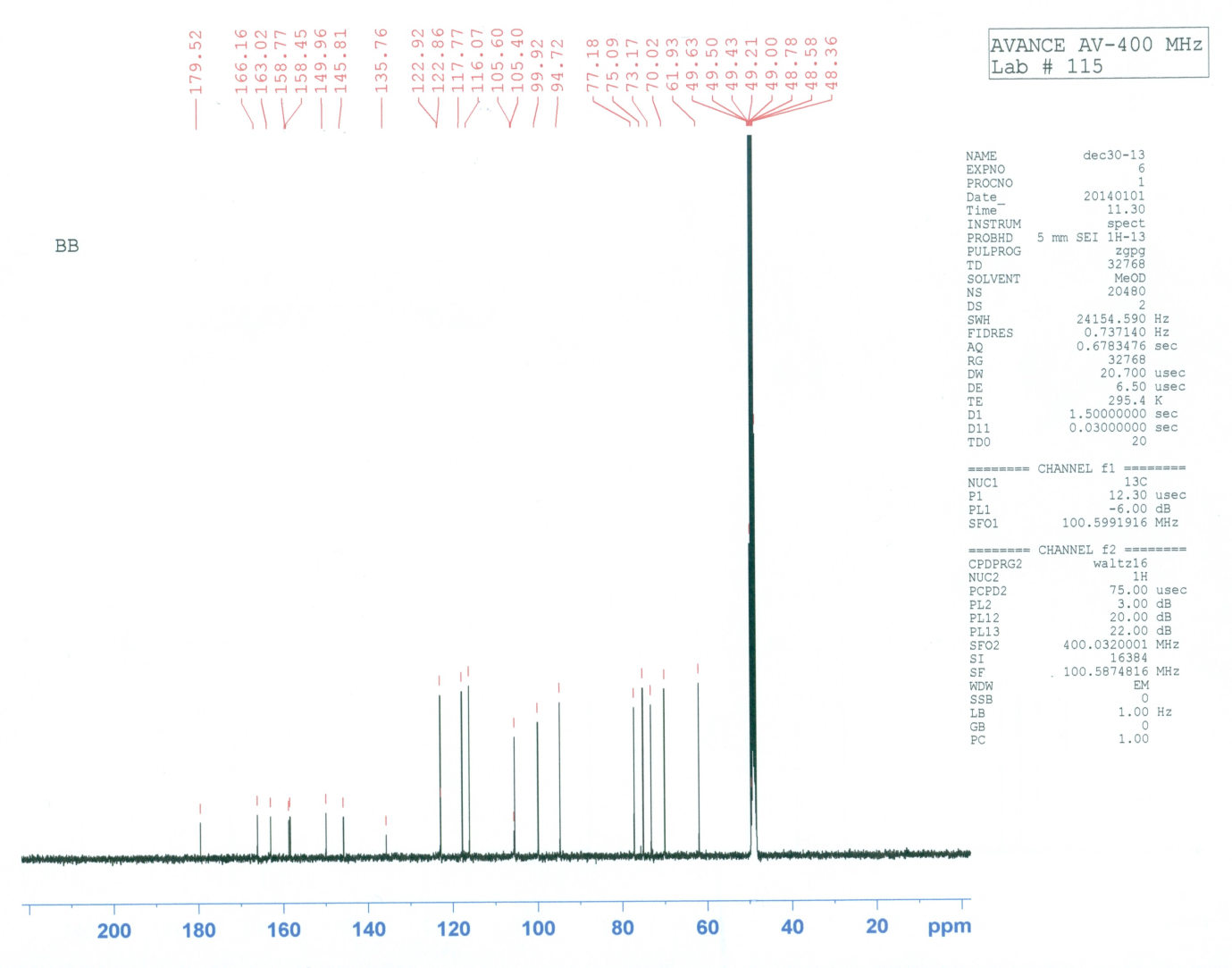
**

**Figure S14:** BB spectrum

**
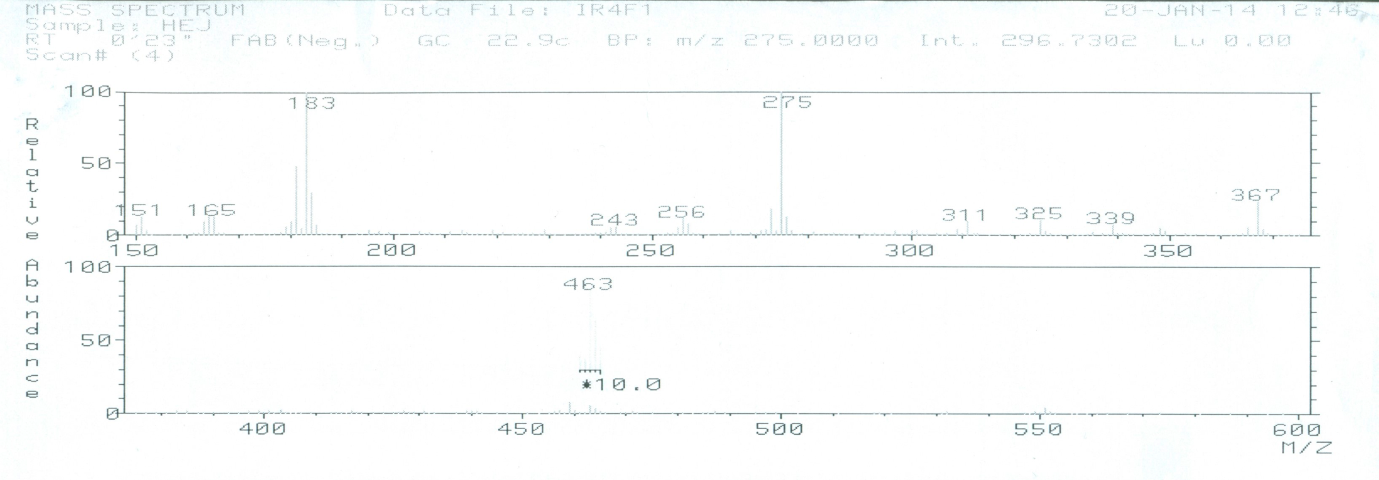
**

**Figure S15:** Mass spectrum

**
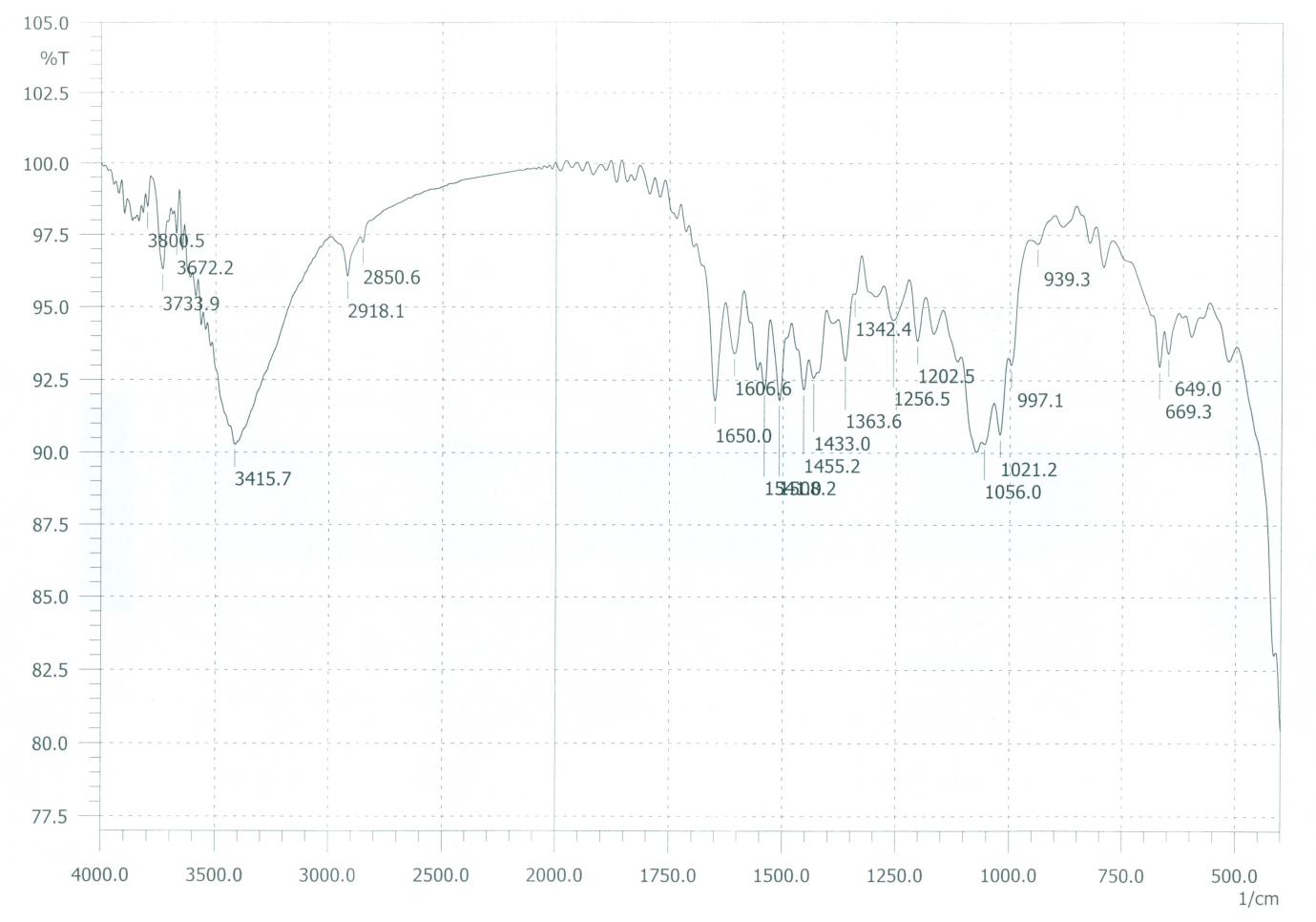
**

**Figure S16:** IR spectrum
